# Topological Entanglement of Linear Catenanes: Knots and Threadings

**DOI:** 10.1021/acsmacrolett.3c00315

**Published:** 2023-08-28

**Authors:** Zahra Ahmadian Dehaghani, Pietro Chiarantoni, Cristian Micheletti

**Affiliations:** International School for Advanced Studies (SISSA), Via Bonomea 265, 34136 Trieste, Italy

## Abstract

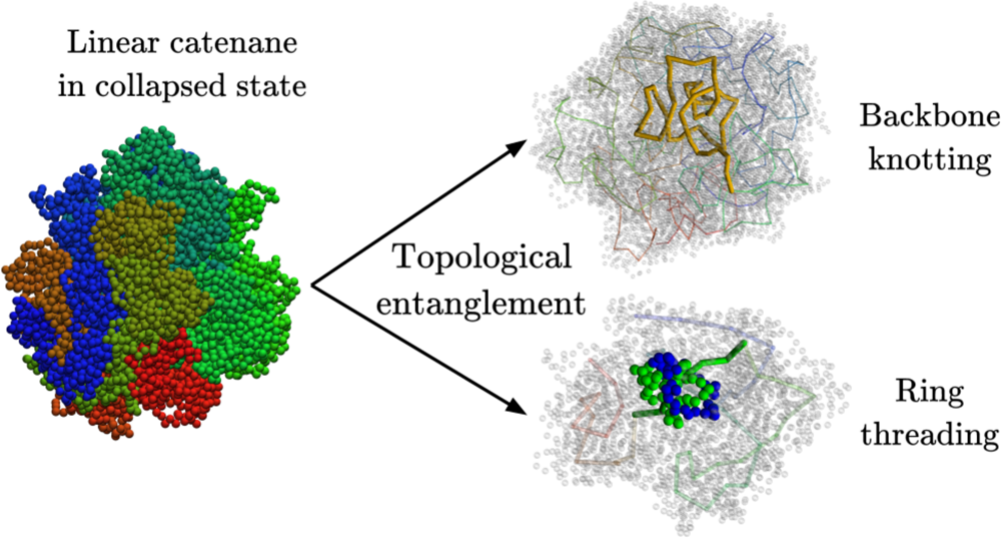

We used molecular dynamics simulations to investigate the self-entanglements of the collapsed linear catenanes. We found two different types of topologically complex states. First, we observed numerous long-lived knotting events of the catenane backbone. However, comparison with conventional polymers reveals that knots are suppressed in catenanes. Next, we observed topologically complex states with no analogue in polymers, where a concatenated ring was threaded by other near or distal rings sliding through it. Differently from knots, these threaded states can disentangle by becoming fully tightened. A detailed thermodynamic and microscopic analysis is employed to rationalize the persistence of threaded states, which can survive significant internal reorganizations of the entire catenane. We finally discuss the broader implications of these previously unreported types of entanglements for other systems, such as noncollapsed and interacting catenanes.

Topological interlockings, also termed mechanical bonds, have become key motifs of supramolecular constructs. They are an essential design element in switchable catalysts,^1−3^ chemically driven motors,^4−6^ self-healing materials,^7,8^ and especially polycatenanes, where several ring polymers are concatenated in a string-like fashion.^9−12^ The overall linear topology of catenanes makes them ideally suited to comparison with conventional polymers, thus contrasting the effects of mechanical and conventional bonding on statics and dynamics. For instance, chains of concatenated rings have been shown to feature nonconventional metric scaling regimes^13−17^ and anomalous relaxation dynamics, both in isolation and in crowded conditions.^18,19^ The mechanical response of catenanes to external perturbations, such as mechanical stretching, translocation, or spatial confinement, is singular, too.^20−24^

However, a key aspect that has remained unexplored for catenanes is whether and how these topologically interlocked constructs can themselves become topologically entangled. These avenues have theoretical and practical implications, given that entanglement in polymers, including physical knots, have been shown to affect the metric and dynamic properties in a broad range of conditions, from spatial confinement to extensional flows to translocation as well as metric and rheological properties.^25−36^

Here, we take the first step in extending this endeavor to polycatenanes. We focus on two main questions. First, how does mechanical bonding affect the knotting of catenanes compared to that of conventional chains? Second, can catenanes establish singular forms of self-entanglement that have no counterpart in linear polymers?

To clarify these questions, we carried out a systematic topological profiling of catenanes of hundreds of rings of varying sizes, which we sampled using molecular dynamics simulations. The catenanes were collapsed with a short-ranged attraction to observe entanglements that would otherwise require impractically large noncollapsed systems to emerge. The setup enabled us to identify long-lived forms of entanglement in catenanes, and reveal their quantitative and qualitative different character compared to conventional polymers.

We considered polycatenanes consisting of *n* = {100, 200, 300, 400} fully flexible interlocked rings, each of *m* = {20, 40} monomers (beads) with diameter σ. Ring connectivity was provided by a standard combination of a repulsive WCA potential and a FENE attraction between consecutive beads.^37^ All other intra- and inter-ring monomer pairs interacted via a smoothly cutoff Lennard-Jones (LJ) potential with amplitude equal to the heat bath thermal energy, ϵ = *k*_B_*T*. The term was modified from the standard LJ one to ensure that the potential and its derivative decay to zero at the cutoff distance *r*_c_, see Supporting Information (SI). The LAMMPS simulation package^38^ was used to evolve the system with stochastic Langevin and Nosé–Hoover dynamics for time scales much longer than the relaxation times at each (*n*, *m*) combination (SI).

For each (*n*, *m*) combination, we gathered 20 or more independent trajectories starting from elongated (straight) catenane configurations. The catenanes were first relaxed with a purely steric interaction (LJ cutoff *r*_c_ = 1.12σ). The cutoff was then extended to *r*_c_ = 1.85σ, in order to include a short-range attraction just sufficient to collapse the chains (SI). The ensuing coarsening dynamics is illustrated in Figure 1, with lumps and dumbells growing and eventually coalescing into a crumpled globule.^39^ The globular catenanes were then evolved for time scales exceeding at least 50 times the nominal relaxation time of the collapsed state, τ, which was obtained from the analysis of the multiscale segmental dynamics of the backbone (SI). Only conformations sampled at simulation times larger than τ were considered to analyze the relaxed collapsed catenanes.

**Figure 1:**
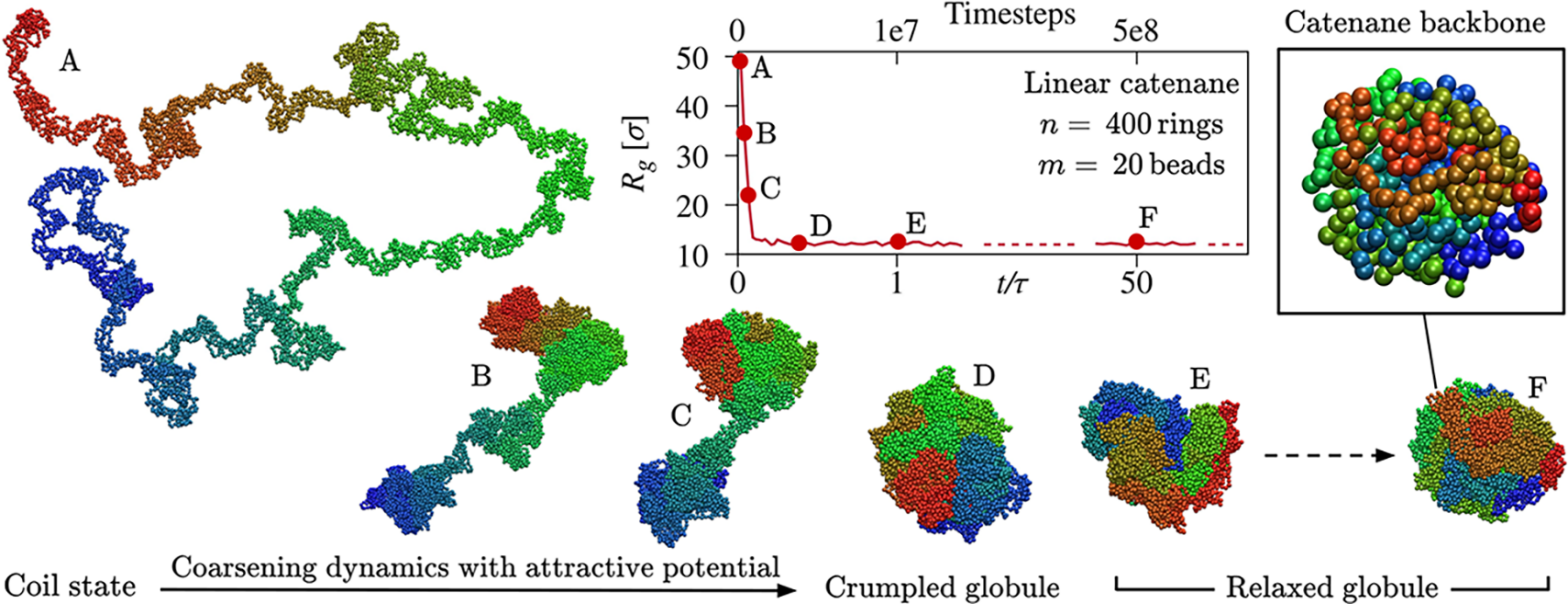
Model and collapsed state. Coarsening dynamics of a catenane of *n* = 400 rings of *m* = 20 beads ensuing after switching on the short-range attractive potential described in the main text. Rings are colored with an end-to-end rainbow scheme. The collapsed catenane, which evolves from the out-of-equilibrium crumpled globule, has a characteristic relaxation time of τ = 1.1 × 10^7^ timesteps of NH molecular dynamics (SI). Only configurations that are nominally relaxed (*t*/τ > 1) were used to characterize the collapsed state. The inset highlights the mechanical backbone, with each bead corresponding to the center of mass of one ring.

Because spontaneous knotting of polycatenanes has not yet been reported, it is difficult to determine a priori how mechanical bonding can affect and bias the knotting propensity of the catenane mechanical backbone, the virtual chain connecting the centers of mass of consecutive rings; see inset in Figure 1. We thus systematically profiled the knots of relaxed collapsed catenanes for 100 ≤ *n* ≤ 400 and *m* = 20. The knotted states were assigned with the kymoknot algorithm,^40^ which is based on standard topological invariants computed for the cognate circular structures obtained by closing the linear backbone with auxiliary arcs.

The topological profiling of the evolving catenanes revealed numerous and long-lasting knotting events. A typical example is given in Figure 2a. The graph establishes three points. First, it provides the heretofore lacking demonstration that knotting can occur in catenanes. Second, the knotting events can be intricate, involving a dynamic buildup of topological complexity, followed by its gradual annihilation. Third, knots’ lifetimes are comparable and can even exceed the catenane relaxation time, τ, purposely used as the temporal axis unit in the graph. Knotting events can be long enough that other prime knots can independently appear in the backbone, resulting in composite knots such as the 3_1_#3_1_ or 3_1_#4_1_ states in Figure 2a. More in general, and analogous to long polymer chains,^41^ changes of knot type occur via the addition or removal of essential crossings,^42^ as in the transitions between 3_1_ and 3_1_#4_1_ or 5_2_ in Figure 2a.

**Figure 2:**
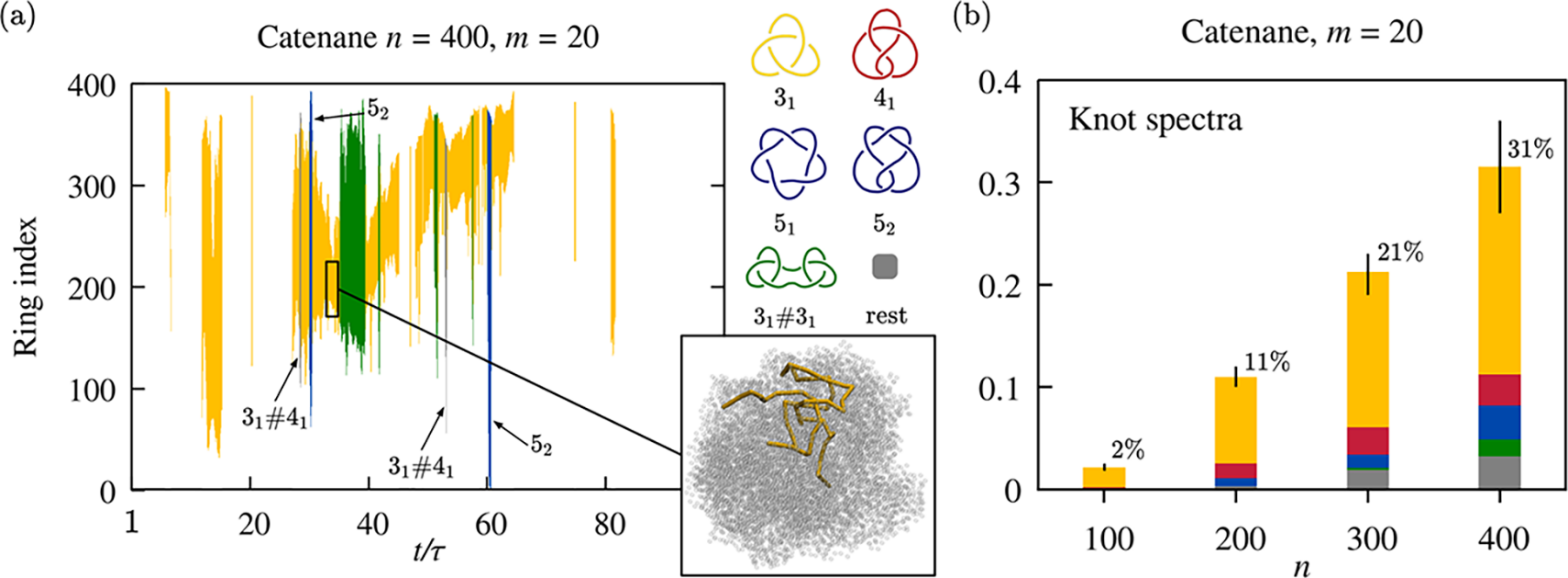
Knotting properties of collapsed catenanes. The kymograph of panel (a) illustrates the typical evolution of the knotted state of a collapsed catenane of *n* = 400 rings of *m* = 20 beads; the bands mark the backbone regions spanned by the knot, which becomes more or less complex with time, as indicated by the color legend. Inset: knotted portion of a catenane backbone. (b) Pile-up histograms of the knot spectrum of collapsed catenanes of *n* rings of *m* = 20 beads. The knotting probability, *p*_*k*_(*n*), is at the top; the bars indicate the estimated statistical error.

Figure 2b presents the knotting probability of the nominally relaxed collapsed catenanes, *p*_*k*_(*n*), which increases rapidly with *n*. We note that these knotting probabilities largely exceed those of just-collapsed (crumpled) catenanes, e.g., 31% versus 2% for *n* = 400 (SI), underscoring that the observed knots in relaxed globular catenanes are not a manifestation of latent entanglements of the initial states, but are genuinely established through spontaneous global changes as shown in Figure 2a. A best fit of the *p*_*k*_(*n*) data indicates that the asymptotic trend is compatible with an exponential decay of the unknotting probability with a characteristic decay length of *n*_0_ = 840 ± 50 rings for the *m* = 20 case (SI).

To establish how catenane knotting differs from conventionally bonded polymers, we compared the *p*_*k*_(*n*) data to the knotting probability of collapsed chains of *n*_b_ beads with bending rigidity equal to the effective one of the catenanes, i.e., 2.2 in simulation units.^17^ The comparison indicates that chains of beads require significantly fewer monomeric units than catenanes to achieve the same level of knotting in the globular state, namely, *n*_b_ ∼ 15.5 + 0.22 *n*; pleasingly, this correspondence extends to the knot spectrum, too (SI). Thus, due to the substantial compenetration of concatenated rings, the knotting of the collapsed catenanes is similar to that of collapsed conventional chains of beads with about 5-fold smaller number of monomer units. We conclude that, under similar conditions, mechanical bonding inhibits knotting compared to conventional bonding.

The second main result of this study is that catenanes can establish long-lived entanglements other than knots and are not accessible to general polymers. These topologically complex states arise when a portion of the catenane deeply threads through one of the concatenated rings, a condition that can be realized thanks to the fact that, despite the overall good packing of the collapsed catenanes, the individual rings are typically not crumpled (SI).

Figure 3 shows the typical evolution of one such threaded state from creation to annihilation. The shown instance and other displayed results are for chains of *n* = 50 rings and *m* = 40 beads. Panel (a) illustrates the stochastic sliding of the backbone through the host ring (index 38). The trace represents the index of the guest ring passing through the host at a given time. The event is initiated by a terminal ring (index 1) entering and passing through ring 38, triggering a long-lasting cascade of other sliding rings (2, 3, etc.).

**Figure 3:**
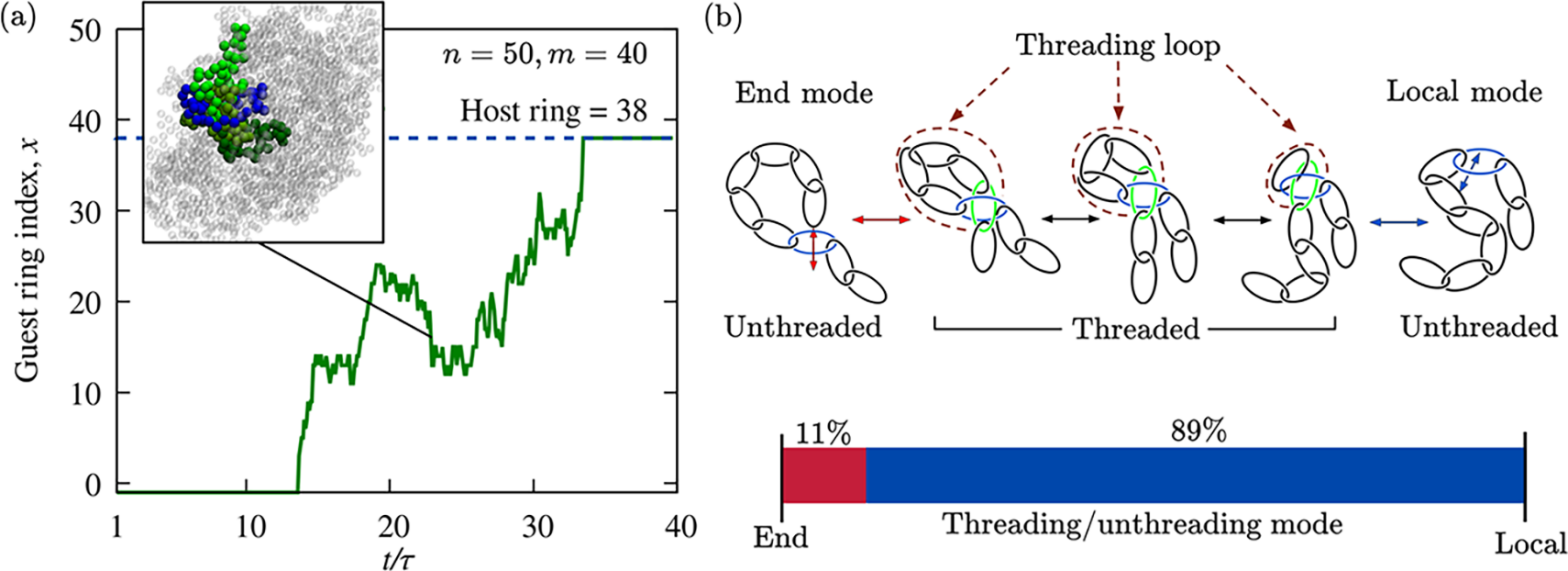
Threaded states formation and annihilation by end and local modes. The typical evolution of a threaded state is shown by the temporal trace in panel (a), representing the instantaneous index of the guest ring threading through the host ring (index 38 in this example). The snapshot shows one configuration of threaded catenane. The host ring is colored blue, while the guest ring and its neighbors are colored green. (b) Schematic illustration of threading creation and annihilation via end and local modes, with a bar histogram representation of their overall incidence in the collected trajectories. Threadings were detected with the method detailed in SI, based on the calculation of the Gaussian integral and an elastic-band smoothing^43^ of backbone-ring pairs.

While it is possible that the threaded state could disentangle by the reverse process, i.e., the opening of the threading loop via the guest backbone retraction, this is not what happens in Figure 3a. The trace reveals that, as time progresses the guest ring approaches the host one, making the threading loop tighter and tighter. Conventional entanglements, such as knots, become more entrenched with tightening. By contrast, the threaded states of catenanes are altogether different because they can be undone by shrinking the threading loop to zero. Figure 3a demonstrates the effect, showing that the catenane disentangles when the host ring (38) is threaded by its next-nearest-neighbor (36). Note that this unthreading mode would not be available to catenanes made of rings with torsional rigidity that are covalently bonded instead of mechanically bonded.

The duration of the considered event surpasses the metric relaxation time by more than 1 order of magnitude. As discussed below, long lifetimes are typical for threadings initiated by a terminal guest ring (end mode). However, threadings can also be established by rings that thread through one of their next-nearest neighbors (local mode). Such processes would, in fact, be the reverse of the annihilation event of Figure 3a.

Over the hundreds of observed threading/unthreading events, we found that the majority (89%) occurred via the local mode. We also observed that all rings in the collapsed catenanes are equally likely to be hosts, except for those near the termini, which are less threaded than average (SI). Overall, nearly 2% of relaxed collapsed conformations for *n* = 50 and *m* = 40 were found to be threaded. By contrast, threadings were virtually absent for *m* = 20, underscoring the necessity of sufficiently large rings for these entangled states to arise.

For a detailed dynamic and thermodynamic characterization, we collected hundreds of Langevin trajectories where a central ring in the collapsed catenane (index 27) was initially threaded at three different depths, corresponding to guest ring indices *x*(*t* = 0) = {4, 14, 23}. The resulting unthreading trajectories are shown in Figure 4a, differently colored for the end (red) and local (blue) disentanglement modes.

**Figure 4:**
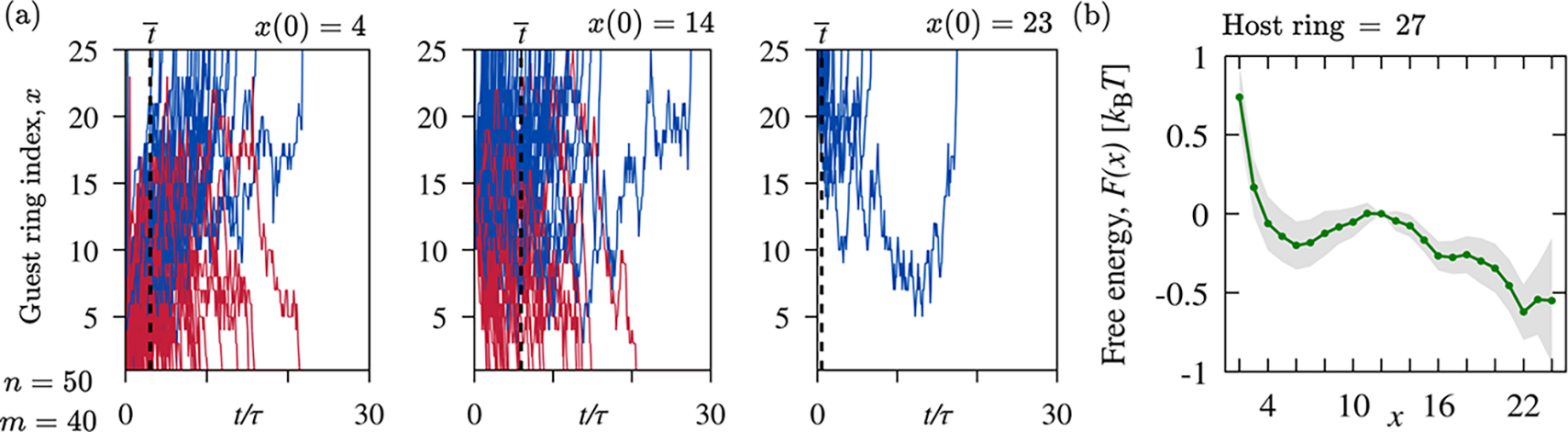
Unthreading dynamics and free energy landscape. (a) The traces are for *n* = 50 and *m* = 40 and illustrate the evolution for three different initial threading depths of ring 27, approximately central in the backbone. For each case, the traces show the evolution of the guest ring index, *x*, over trajectories and are differently colored for the end (red) and local (blue) disentanglement modes. (b) Free-energy landscape inferred from the traces of panel (a); the estimated error is shown by the shaded region.

The average lifetimes (dashed line) of threadings started near the termini, *x*(0) = 4, and near the host, *x*(0) = 23 are equal to 3τ and 0.5τ, respectively. The longer duration of the former indicates that entanglements initiated by host–guest pairs that are well separated along the backbone typically outlive significant geometric reorganizations of the collapsed catenane, as already noted in connection with Figure 3.

To recover the thermodynamic potential underpinning the observed trajectories, we modeled the stochastic displacements of the guest ring index, Δ, taken at time intervals d*t* = 0.09τ, as a one-dimensional Wiener process with uniform diffusion coefficient^44^ (SI). For such processes, the thermodynamic force at a given position *x̅* can be determined from the local drift and diffusive terms,^45^ respectively, proportional to  and , where the Kramers-Moyal  brackets denote averages restricted to displacements originating at position *x̅*. For our system, where the variable *x* is the guest ring index and hence is adimensional, the reduced thermodynamic force at *x̅* is given by , where var(Δ) is the average of the diffusive term taken over the entire guest ring range, as appropriate for a uniform diffusion coefficient (SI). The free energy landscape, *F*(*x*), is then recovered by numerical integration of the thermodynamic force.

Figure 4b presents the resulting *F* profile, reconstructed in the entire *x* range except close to the annihilation points *x* = 1 and 25. The profile is noticeably asymmetric with respect to the local maximum for *x* = 11, taken as the *F* = 0 level, corresponding to the guest ring being halfway between the annihilation points. Moving away from this midpoint, the potential decreases to −0.5*k*_B_*T* for *x* → 25, whereas it exhibits a minimum at *x* = 6 and then increases more steeply for *x* → 1. This clarifies that unthreading via the end mode is hindered by a free-energy barrier of order *k*_B_*T*, while it is unimpeded via the local mode, consistent with the different characteristic lifetimes discussed above. Furthermore, away from the annihilation points, the *F* landscape is relatively flat, allowing a diffusive-like sliding motion of the guest segment. This explains why threaded states originating from both modes can persist for a long time after approximately five rings have threaded through the host ring, as shown in Figure 4.

The microscopic origin of the free energy barrier for *x* → 1 is clarified by the relative spatial positioning of the rings in the globule. We found that terminal and host rings tend to segregate, occupying the surface and inner core of the collapsed chain, respectively. These opposite different biases are conveyed by the radial probability distributions of Figure 5, which were computed over all threaded conformations sampled in the relaxed collapsed state and hence for varying indices of the host rings.

**Figure 5:**
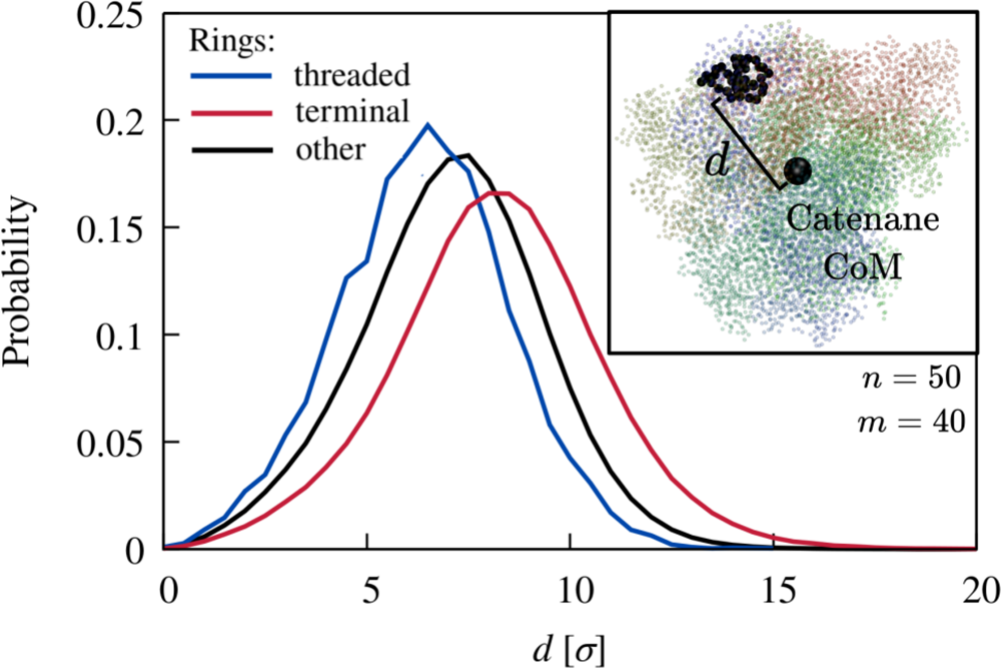
Radial positioning of terminal, host, and other rings. Probability distribution of the distance of the centers of mass (CoM) of host, terminal, and other rings from the CoM of the globule, with an illustrative configuration shown in the inset.

We explained the radial biases by examining branched polymers, which served as a simplified model system, given that threadings can be considered as branching points for the catenanes’ backbone. We found that collapsed branched polymers have the same biases observed in catenanes, with termini preferentially at the surface and branching points at the core (SI). The result highlights the role of effective ring connectivity in catenanes, with terminal rings, which have lower-than-average connectivity, staying at the surface, while threaded rings, with higher-than-average effective connectivity, are drawn to the core. We thus conclude that the free-energy barrier for *x* → 1 reflects the entropic cost of pulling the exposed terminal ring through the buried host one.

To conclude, we studied the reversible formation of distinct types of topologically complex states in collapsed polycatenanes. First, we reported on the spontaneous knotting and unknotting of the catenane backbone while also showing that it is much suppressed compared to the corresponding collapsed states of conventional chains, a fact that explains, at least in part, why catenane knotting has not been observed so far. A second type of entanglement, unique to catenanes, was observed, arising from one of the concatenated rings threading through another. These threaded states, like knotted ones, can be significantly long-lived. Unlike knots, catenane threadings have not one but two annihilation pathways of which we analyzed the thermodynamics: they can be untied by opening up the threading loop and by fully closing it. A relevant avenue for future study would investigate how the rings’ bending and torsional rigidity affect the catenane compliance to knotting and threading. The findings have broader implications for other systems, such as dense solutions or melts, where intercatenane threadings can be expected for sufficiently large rings. Being long-lived, these threadings could, in principle, accumulate, leading to novel collective states created by the networks of persistent, though reversible, interlockings. Finally, we note that the growth of the looped catenane backbone via the (passive) local mode is reminiscent of models for (active) loop-extrusion in chromatin, and thus a suitable comparative study of the two systems might be helpful to model different physical mechanisms concurring to chromatin organization.^46^

## Supporting Information Available

Containing additional details about the model and system setup, the definition of corresponding linear chains, the statics and dynamics of collapsed catenanes, and the detection of their entangled states (PDF)
