# Supplementary material for: Topological Entanglement of Linear Catenanes: Knots and Threadings
Source: ACS Macro Lett. 2023 Aug 28;12(9):1231–6. doi: 10.1021/acsmacrolett.3c00315 (PMC10515615; doi:10.1021/acsmacrolett.3c00315)
Supplement: Supplementary file 1 — mz3c00315_si_001.pdf [file mz3c00315_si_001.pdf]

Supporting Information

"Topological entanglement of linear catenanes: knots and threadings"

Zahra Ahmadian Dehaghani,[1] Pietro Chiarantoni,[1] and Cristian Micheletti[1]
[1] *International School for Advanced Studies (SISSA), Via Bonomea 265, 34136 Trieste, Italy*
(Dated: August 25, 2023)

MODEL AND SIMULATION SETUP

We considered polycatenanes of $n = \{100, 200, 300, 400\}$ fully-flexible interlocked rings, each of $m = 20$ or 40 monomers (beads) with diameter σ. Bonded-monomers in each ring interacted with a standard combination of a finitely-extensible nonlinear elastic (FENE) potential and a truncated and shifted Lennard-Jones (WCA) term, $U_{\rm FENE} + U_{\rm WCA}$, with

$$U_{\rm FENE}(r) = -0.5KR_0^2 \ln\left[1 - \left(\frac{r}{R_0}\right)^2\right] \tag{S1}$$

$$U_{\rm WCA} = \begin{cases} 4\epsilon[(\frac{\sigma}{r})^{12} - (\frac{\sigma}{r})^6 + \frac{1}{4}] & r \le 2^{1/6}\sigma \\ \\ 0 & \text{otherwise,} \end{cases} \tag{S2}$$

where $R_0 = 1.5\sigma$, $k = 30.0\epsilon/\sigma^2$. The WCA potential amplitude was set equal to the system thermal energy, $\epsilon = k_BT$.

All other pairs of intra- and inter-ring monomers interacted via a smoothly-cutoff Lennard-Jones potential,

$$U(r) = \begin{cases} U_{LJ}(r) - U_{LJ}(r_c) - (r - r_c)\left.\frac{dU_{LJ}}{dr}\right|_{r=r_c} & r \le r_c \\ \\ 0 & \text{otherwise,} \end{cases} \tag{S3}$$

where $U_{LJ}(r) = 4\epsilon[(\frac{\sigma}{r})^{12} - (\frac{\sigma}{r})^6]$, and the cutoff r_c was varied in the $[1:3.5]\sigma$ range to locate the collapse transition, as detailed further below. For $r_c = 2^{\frac{1}{6}}\sigma \simeq 1.122\sigma$ the potential reduces to the purely repulsive WCA one. For larger r_c's the potential includes a short-range attraction.

The LAMMPS simulation package[1] was used to perform molecular dynamics simulations. Local (Langevin) and global (Nosé-Hoover) thermostats were used to characterize the catenanes' dynamics and statics, respectively. Simulation parameters, such as friction coefficients and monomers' mass (M) were set to standard values[2]. The integration time steps of Nosé-Hoover (NH) and Langevin dynamics (LD) were set equal to $\Delta t = 0.005\tau_{\rm LJ}$, with $\tau_{\rm LJ} = \sigma(\epsilon/M)^{1/2}$. The Nosé-Hoover temperature damping parameter was set to $t_{\rm damp} = 0.5\tau_{\rm LJ}$. For each considered (n, m) combination we gathered at least 20 trajectories, each covering time spans from $1 \cdot 10^7$ $\tau_{\rm LJ}$ ($n = 100$) to $3 \cdot 10^6 \tau_{\rm LJ}$ ($n = 400$).

LINEAR CHAINS OF BEADS

The knotting probability of collapsed catenanes made of n rings with $m = 20$ beads was compared to that of collapsed chains of n_b beads of size σ and with bending rigidity equal to the effective one of the catenanes, i.e. 2.2 in simulation units[3]. Bonded monomers interacted via the FENE and WCA terms of eqs. S1 and S2, while non-bonded ones interacted via the same smoothly-cutoff LJ-like potential of eq. S3, with r_c chosen as described further below. The knotting probability of the relaxed state of the collapsed chains of beads was studied with NH molecular dynamics simulations at different values of n_b in the 25–112 range. Five independent trajectories were collected for each value of n_b, each of duration ranging from $2 \cdot 10^6 \tau_{\text{LJ}}$ ($n_b = 25$) to $7 \cdot 10^5 \tau_{\text{LJ}}$ ($n_b = 112$).

COLLAPSED STATE

To identify the collapse transition, we analyzed the average gyration radius, R_g, of catenanes ($n = 100, m = 20$) and chains of beads ($n_b = 100$) for different values of r_c, the interaction range of the smoothly-cutoff Lennard-Jones potential, eq. S3. The R_g curves, shown in Fig. S1, present inflection points in correspondence of the crossover from coil to collapsed state. We considered r_c values just below the collapse transition, namely $r_c = 1.85$ for catenanes and $r_c = 2.1$ for the chains of beads.

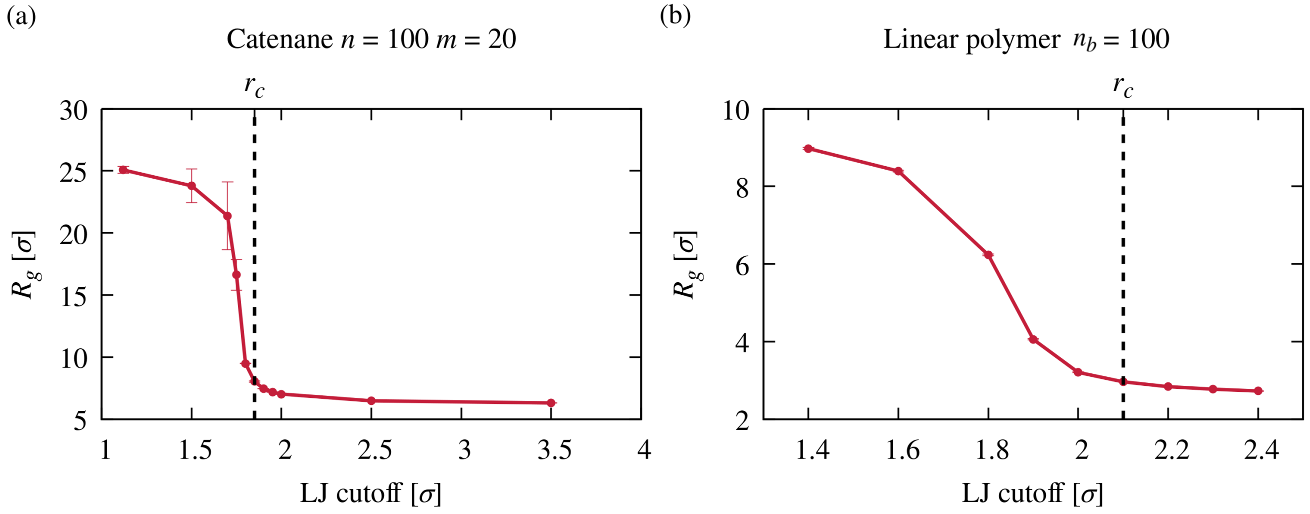

FIG. S1. **Average gyration radius of ($n = 100, m = 20$) catenanes with and chains of beads chains as a function of the interaction range cutoff distance, r_c.** The dashed lines mark the r_c values selected to study the collapsed states of the two systems.

RELAXATION TIMES

We analyzed the relaxation dynamics of segments of increasing length, $|i - j|$ of the collapsed catenanes. For each segment length, we computed the average root-mean-square distance (RMSD) of optimally-superposed (Kabsch-aligned) backbone segments at different time-lags. This timescale quantifies the typical time required by a portion $|i-j|$ of the catenane backbone to reorganize internally, i.e. discounting global rotations and translations. The results for different (n, m) combinations are shown in Fig. S2.

We then identified the relaxation time associated to each curve as the lag for which the RMSD reaches 90% of the plateau value. Fig. S3 shows the results as a function of the segment length and for different (n, m) combinations. As intuitively expected, the slowest relaxation is observed for the longest segments, $|i - j| = n$, and the corresponding timescale, τ, is thus taken as the relaxation time of the collapsed catenane.

For comparison, Fig. S3 also shows the curves of the rotational relaxation times of the segments, $\tau_{\text{rot},|i-j|}$, obtained

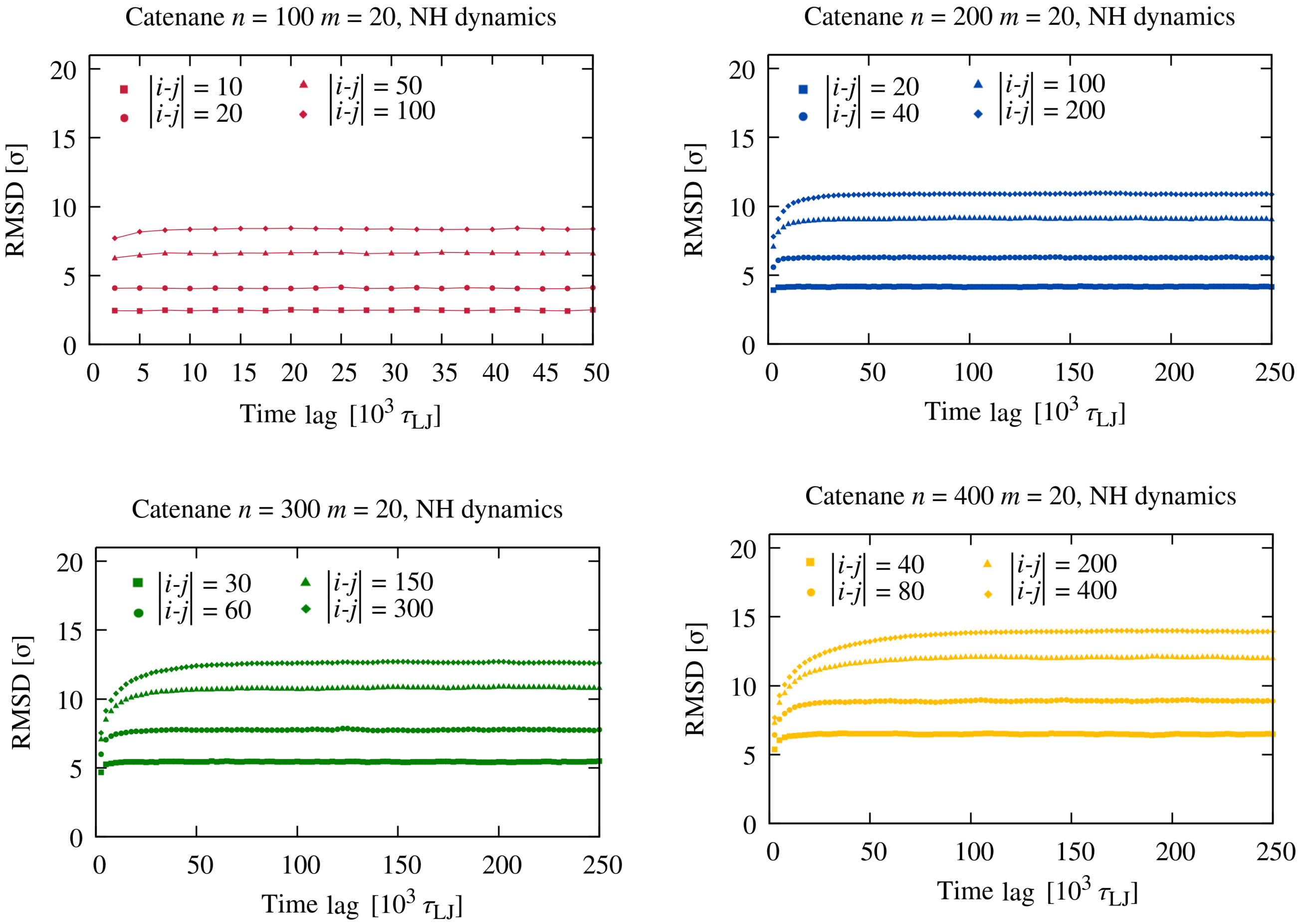

FIG. S2. Root mean squared distance of optimally aligned backbone segments of length $|i-j|$ at different time lags. For each (n, m) combination, data are averaged over all backbone segments of length $|i-j|$.

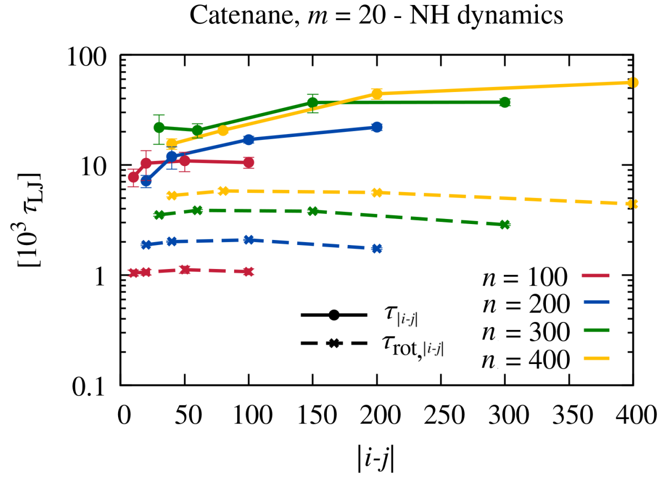

FIG. S3. Comparison of $\tau_{\mathrm{rot},|i-j|}$ and $\tau_{|i-j|}$ for different segment lengths $|i-j|$ for each $(n.m)$ combination.

by numerical integration of the correlation function of normalized end-to-end vector of the catenane backbone, $\vec{u}(t)$,

$$\tau_{\rm rot} = \int_0^{\bar{t}} d\tau\, C_{\rm rot}(\tau) \ , \qquad \text{with} \ \ C_{\rm rot}(\tau) = \langle\langle \vec{u}(t)\cdot\vec{u}(t+\tau)\rangle_t\rangle \ . \tag{S4}$$

In the expression above, the double brackets indicate averaging over time and over simulations and $\bar{t}$ is the shortest lagtime for which C drops below 10^{-2}.

Tab. S1 summarizes the relaxation times, τ, and the rotational ones, $\tau_{\rm rot}$, for the entire collapsed catenane. The data shows that in the collapsed state the rotational dynamics is at least one order of magnitude faster than the internal reorganization dynamics.

Catenane in collapsed state

n	m	MD thermostat	$\tau_{\rm rot}/\tau_{\rm LJ}$	$\tau/\tau_{\rm LJ}$
100	20	NH	1072 ± 63	$(10.5 \pm 1.2) \cdot 10^3$
200	20	NH	1736 ± 27	$(22.0 \pm 1.4) \cdot 10^3$
300	20	NH	2863 ± 52	$(37.1 \pm 2.6) \cdot 10^3$
400	20	NH	4418 ± 140	$(56.1 \pm 2.7) \cdot 10^3$
50	40	NH	3838 ± 45	$(18.8 \pm 1.7) \cdot 10^3$
50	40	LD	7200 ± 302	$(29.4 \pm 3.9) \cdot 10^3$

TABLE S1. **Relaxation times.** Characteristic internal reorganization times, τ, and rotational times of the end-to-end vector, $\tau_{\rm rot}$, of collapsed catenanes for different (n, m) and thermostats combinations. LD and NH stand for Langevin and Nosé-Hoover Dynamics, respectively.

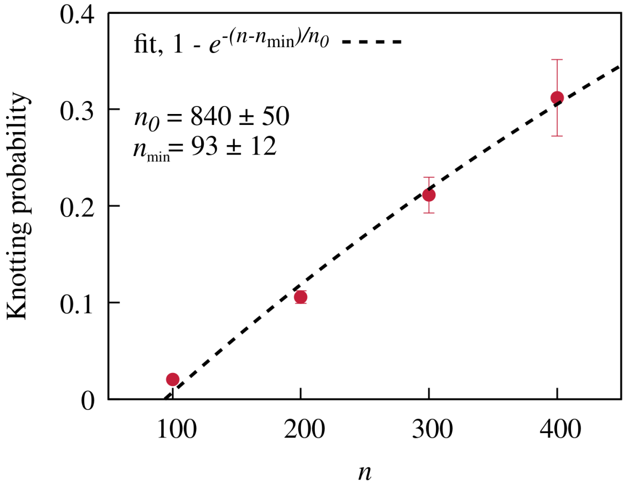

FIG. S4. **Knotting probability of collapsed catenanes.** Knotting probability of the collapsed catenane backbone as a function of the number of rings n for $m = 20$. The dashed line is the best fit of the data with the functional form $p_k(n) = 1 - \exp[-(n - n_{\min})/n_0]$. The best fit parameters are $n_{\min} = 93 \pm 12$ and $n_0 = 840 \pm 50$.

TOPOLOGICAL ANALYSIS

Knotting of relaxed collapsed catenanes

Physically-knotted states of the catenane backbone were identified and located using the kymoknot package[4] which employs a minimally invasive scheme to close the backbone into a ring. The proper topological state of the closed backbone was then established by computing the Dowker code and comparing it against look-up tables for prime knots up to 16 crossings available as part of the knotscape package developed by Jim Hoste and Morwen Thistlethwaite. Factorizations of the Dowker code further allowed to identify composite knots.

In the kymoknot package, the closure scheme is combined with a bottom-up search of the shortest knotted region to yield the location of knot on the backbone contour. To suppress noise, we assigned the knot type based on a majority rule applied to snapshots covering intervals of $1.7 \times 10^4 \tau_{\rm LJ}$. A running average over the same time intervals was used to assign the knot boundaries.

The knotting probability p_k for catenanes of $n = 100, 200, 300, 400$ rings of $m = 20$ beads is shown in Fig. S4. The solid line is the best fit of the data based on an exponential decay of the unknotting probability, $p_k(n) = 1 - \exp[-(n - n_{\min})/n_0]$. The fitting parameter $n_{\min}$ accounts for the fact that, due to finite size effects, a minimum catenane length is needed to observe knotting. The other parameter, n_0, is the exponential decay length of the unknotting probability. The best fit of the data yields $n_{\min} = 93 \pm 12$ and $n_0 = 840 \pm 50$.

Knotting of just-collapsed (crumpled) catenanes

For contrast with the relaxed globular catenanes, we measured the knotting probability of catenanes just after collapse, when they are still in the out-of-equilibrium crumpled state.

To this end, we followed the collapse process of catenanes of $n = 400$ rings of $m = 20$ beads starting from 500 independent initial configurations, corresponding to the equilibrium coil state (no self-attraction). The plot of Fig. S5 shows the evolution of the gyration radius, R_g for the 500 trajectories. For each trajectory, the just-collapsed state was defined by the earliest instance when the R_g fell below the average value of the relaxed collapsed state, indicated by the dashed line in the figure.

The knotting probability observed over the ensemble of the 500 crumpled catenanes was equal to 2.4 ± 0.5 %, hence an order of magnitude smaller than in the relaxed collapsed state.

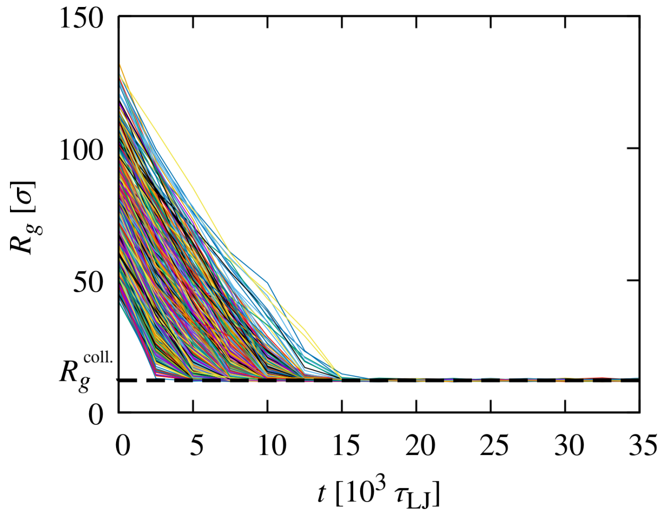

FIG. S5. **Selection of just-collapsed catenanes.** Time evolution of the gyration radius of catenanes from 500 initial coil states after switching on the short-range attractive potential. For each trajectory, the just-collapsed state was defined by the earliest instance when the R_g fell below the average value of the relaxed collapsed state, indicated by the dashed line in the figure.

Comparison with knotting of collapsed chains of beads

We compared the knotting of collapsed catenanes with that of the collapsed linear chains of n_b beads described on page 2.

The length dependent knotting probability for the conventional collapsed chains is shown in Fig. S6a. The horizontal dashed lines in the same plot mark the knotting probabilities of collapsed catenanes of various number of rings of $m = 20$ beads. The intersection of the horizontal lines with the chains of beads knotting probability (piecewise-linear interpolation) establishes a correspondence between the number of rings, n, and the number of beads, n_b, in the two

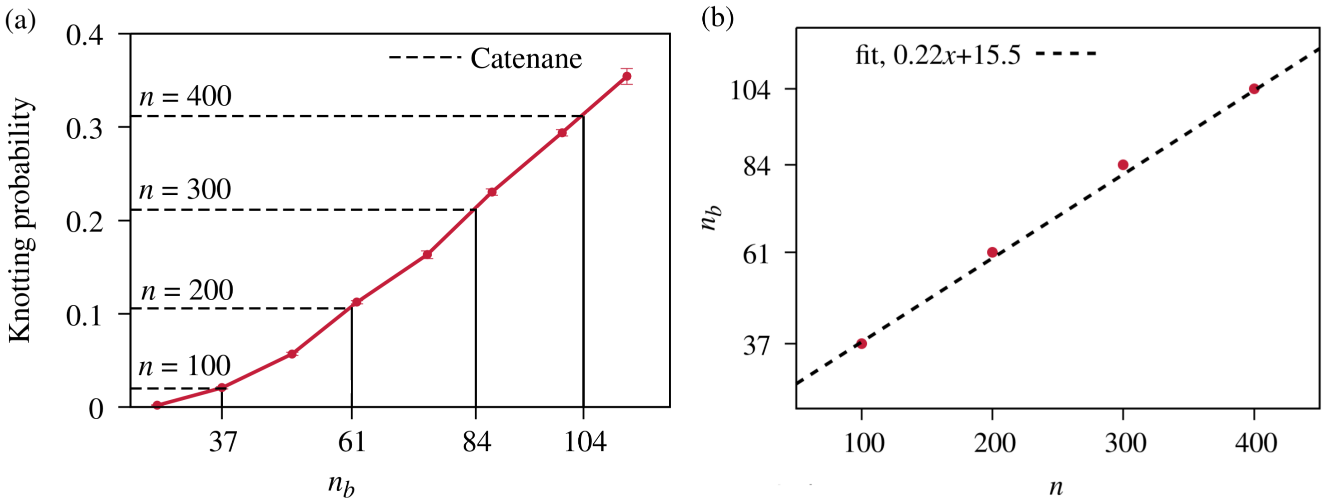

FIG. S6. **Comparison of knotting probabilities of collapsed catenanes and chains of beads.** Panel (a): knotting probability of collapsed chains of n_b beads. The horizontal dashed lines mark the knotting probability of collapsed catenanes of $n = \{100, 200, 300, 400\}$ rings of $m = 20$ beads. The intersection of the horizontal lines with the chains of beads knotting probability (piecewise-linear interpolation) establishes a correspondence between the number of rings, n, and the number of beads, n_b, which is shown in panel (b). The linear best fit of the corresponding data (dashed line) yields: $n_b = a + b\,n$, with $a = 15.5 \pm 1.8$ and $b = 0.22 \pm 0.01$.

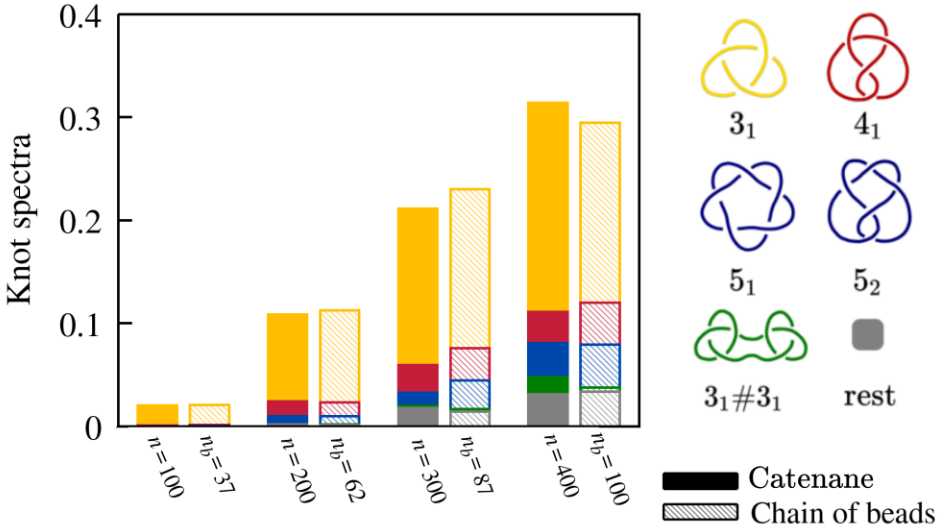

FIG. S7. **Knot spectra for collapsed catenanes and linear chains of beads.** Pileup histograms for the knot spectrum of collapsed catenanes (saturated colors, solid bars) and chains of beads (transparent colors, hatch-filled bars) of approximately corresponding number of rings (n) and beads (n_b).

systems. This correspondence is explicitated in Fig. S6b, where the dashed line is the linear best fit of the data, corresponding to $n_b \sim 15.5 + 0.22n$.

The correspondence also extends to the knot spectra, as shown in Fig. S7.

Threadings

To detect threadings in a given catenane configuration, we considered each ring of the catenane in turn, $i = \{1, ..., n\}$, and tested whether it was threaded or not by the two arms of the catenane departing from it, $[1 : i - 2]$ and $[i + 2 : n]$ (nearest-neighbor rings, $i \pm 1$, were not considered as they are concatenaned to ring i). Each arm was then turned into a polymer chain by connecting suitable portions of the rings. For instance, starting from ring $k = i + 2$, we would first identify its two monomers closest to those of the preceding and following rings, $j_{k,k-1}$ and $j_{k,k+1}$. We would then take the shortest segment of the ring (from $j_{k,k-1}$ to $j_{k,k+1}$, or vice-versa) as the first leg of the auxiliary polymer chain. We next connected the end monomer of this first leg, $j_{k,k+1}$, to the first monomer of the second leg, $j_{k+1,k+2}$, see sketch in Fig.S8a. We note that the closest monomers of subsequent rings are typically at a distance of order σ in the collapsed state, see Fig. S8b.

After completing the construction of the two arms, we checked whether any one of them was threading ring i. To do so, we first computed the Gauss integral of the ring and each of the arms,

$$\frac{1}{4\pi} \oint_{\text{ring}} \int_{\text{arm}} \frac{\vec{r}_1 - \vec{r}_2}{|\vec{r}_1 - \vec{r}_2|^3} \cdot (d\vec{r}_1 \times d\vec{r}_2) \tag{S5}$$

and discarded from further considerations cases where the integral returned values smaller than 0.65 in absolute value, a conservative threshold for unthreaded states. A stringent and more computationally intensive threading test was carried out on the remaining ring-chain pairs. Both the ring and the auxiliary chain were iteratively shrunk and smoothed using a procedure analogous to that of ref[5], disallowing crossings between the ring and the chain. At the end of this procedure, properly threaded state present tightly-contacting ring and chain monomers, which are absent in non-threaded states, see Fig. S8c,d. In case of threadings, the indices of the auxiliary chain monomers in tight contact with the (smoothed) ring allowed us to identify the guest ring index. Finally, noise in the threaded state evolution was eliminated with the same majority rule of the knotted state, applied to running batches covering a duration of $1.2 \times 10^4 \tau_{\text{LJ}}$.

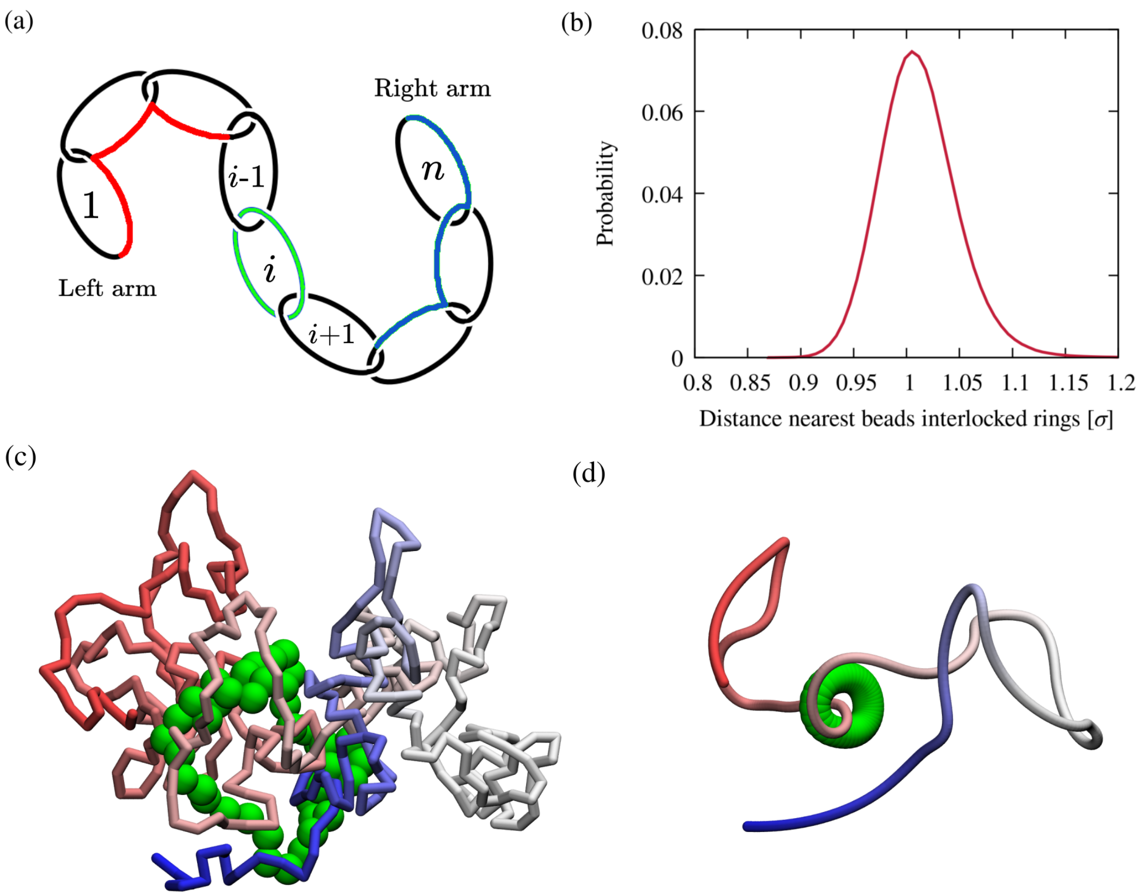

FIG. S8. **Detection of threaded states.** (a) To establish whether ring i is threaded by the preceding (left) or following (right) arm of the catenane, we first construct a linear chain for each of the two arms, connecting suitable portions of the rings, as sketched in the figure and described in the text. Consecutive rings are connected in correspondence of their nearest monomers, which typically are at a distance of order σ, see histogram in panel (b). For unthreaded states, as in the sketched example, the Gauss Linking number of any ring i with the left and right arms is appreciably smaller than one. Configurations with large values of the linking number (> 0.65), as in panel (c), are passed to a more stringent test, where the arms and the putative host ring are shrunk and smoothed, so that the threaded state can be confidently established, as shown in panel (d) .

Fig. S9 illustrates the equilibrium probability for ring $i = \{1, ..., n\}$ to host a threading. The probability profile is for catenanes with $n = 50$ rings and $m = 40$ beads per ring. The profile is uniform except near the termini, where the probability drops approximately by a factor of 2. Overall, 1.76% of the sampled configurations for $n = 50$ rings and $m = 40$ were threaded. We also observed hundreds of doubly-threaded states, as shown in Fig. S10. The probability of such states is 0.04%, which is close to the square of the single-threading probability.

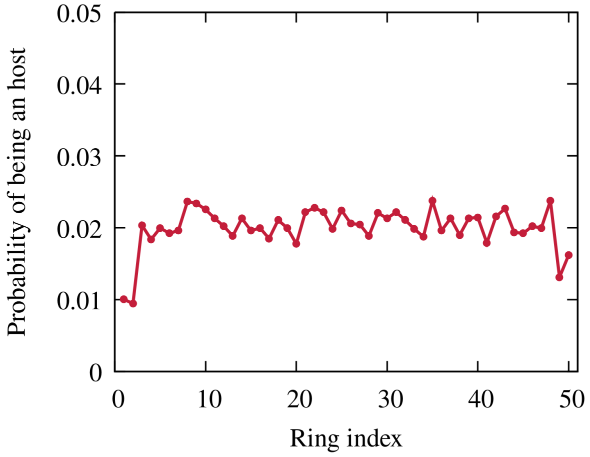

FIG. S9. **Equilibrium probability to host threadings.** The profile shows the probability that rings in ($n = 50, m = 40$)-catenanes are hosts of threaded states. The distribution is uniform along the backbone except near the ends.

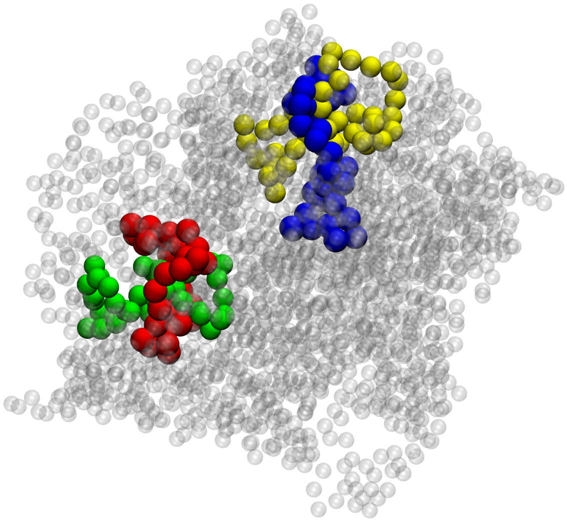

FIG. S10. **Snapshot of a double threading event.** A rare occurrence was observed where two rings were simultaneously threaded. The snapshot illustrates ring 33 (highlighted in red) threaded by ring 43 (highlighted in green), and ring 37 (highlighted in blue) threaded by ring 5 (highlighted in yellow).

CALCULATION OF $\langle\Delta\rangle$

The free energy profile of the guest index x presented in main text Fig. 4b ($n = 50, m = 40$, host index $= 27$) was reconstructed using the maximum-likelihood approach of ref.[6], devised for discrete-time (Wiener) stochastic processes. For the one-dimensional case, such as the evolution of the host ring index, x, a Wiener process can be written:

$$dx_t = v(x_t)\,dt + \sqrt{2\,D(x_t)}\,dW_t \tag{S6}$$

where the subscripted time t is discretized in steps equal to dt, $dx_t = x_t - x_{t-1}$ is the displacement of the variable at time t, D is a position-dependent diffusion coefficient, dW is a Wiener noise term (average equal to zero and variance equal to dt) and $v(x)$ is a position-dependent drift term defined by $v(x) = \partial_x D(x) - \frac{D(x)}{k_B T}\partial_x F(x)$, with $F(x)$ the free energy profile. From this dynamics one can compute the drift and the diffusion terms as

$$v(\bar{x}) = \frac{\langle dx \rangle_{\bar{x}}}{dt}, \qquad D(\bar{x}) = \frac{\langle dx^2 \rangle_{\bar{x}} - \langle dx \rangle_{\bar{x}}^2}{2dt}, \tag{S7}$$

where $\langle \rangle_{\bar{x}}$ indicates the average of sampled displacements dx originating at the index $\bar{x}$.

Fig. S11 shows the probability distributions of the displacements sampled across the collected unthreading trajectories using a sampling time interval $dt = 2.5 \times 10^3 \tau_{\rm LJ}$. The Gaussian-like distributions are grouped in different panels according to the initial guest ring index. For initial positions close to the boundaries, a fraction of the displacements directly lead to unthreading, a fact that is reflected by the distributions being truncated and smaller than average height (because unthreadings are still counted in the normalization). Except for the cases near the boundaries, the distributions have about the same variance, which on average is 1.6. The uniform variance indicates that the diffusion coefficient of the model Wiener process can be considered uniform, too. With this proviso, the Wiener process can be written more simply as:

$$dx_t = -\frac{D}{k_B T} \partial_x F(x)\, dt + \sqrt{2\, D}\, dW_t \ , \tag{S8}$$

from which the free energy $F(x)$ is obtained as[6]:

$$\frac{F(x)}{k_B T} = -\frac{2 \sum_{\bar{x}} \langle \Delta \rangle_{\bar{x}}}{\langle \langle \Delta^2 \rangle_{\bar{x}} - \langle \Delta \rangle_{\bar{x}}^2 \rangle} = -\frac{2 \sum_{\bar{x}} \langle \Delta \rangle_{\bar{x}}}{\mathrm{var}(\Delta)} \tag{S9}$$

where $\mathrm{var}(\Delta) = 1.6$, based on the uniformity noted before, and the $\langle \Delta \rangle_{\bar{x}}$ values were obtained as the midpoint of the best-fitting Gaussians to the observed displacements in Fig. S11; the Gaussians were constrained to have variance equal to 1.6.

Finally, we verified *a posteriori* the applicability of eq. S8, by considering excerpts of unthreading trajectories with x ranging in $[6, 16]$, where the free-energy landscape is approximately flat. We then computed the time autocorrelation function of x, shown in Fig. S12, finding that it becomes practically negligible in one sampling step, $dt = 2.5 \times 10^3 \tau_{\rm LJ}$, consistent with the overdamped nature of the Wiener process.

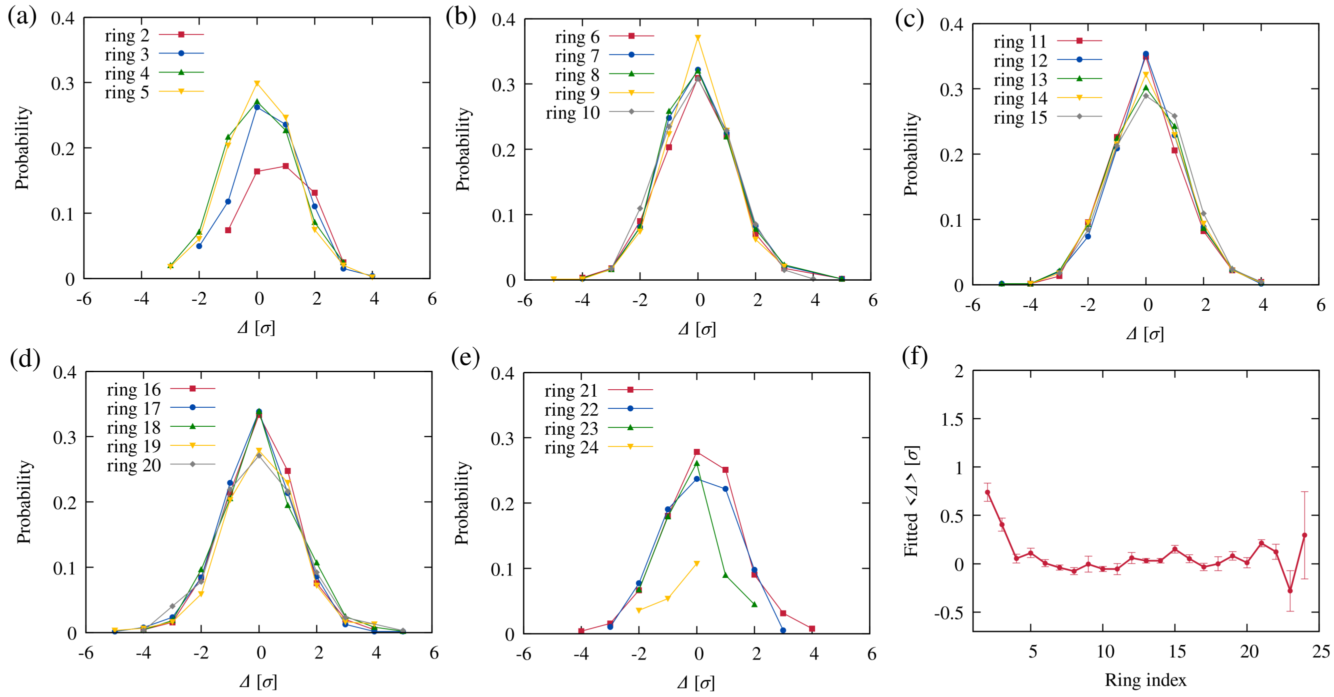

FIG. S11. **Threading displacements.** Panels (a)-(e) show the probability distributions of the observed displacements (changes in guest ring index) in a time interval $dt = 2.5 \times 10^3 \tau_{\rm LJ}$ starting from different positions (indices) of the guest rings. The displacements starting close to the termini have a truncated distribution due to the catenane becoming unthreaded. As revealed by the good overlap, the distributions have about the same (dimensionless) variance equal to 1.6, indicating that the threading motion can be modelled as a stochastic process with uniform diffusion coefficient. The average displacements for each initial position were thus obtained as the mid-point of the best-fitting Gaussians with fixed variance equal to 1.6. The resulting profile of the average displacements is shown in panel (f).

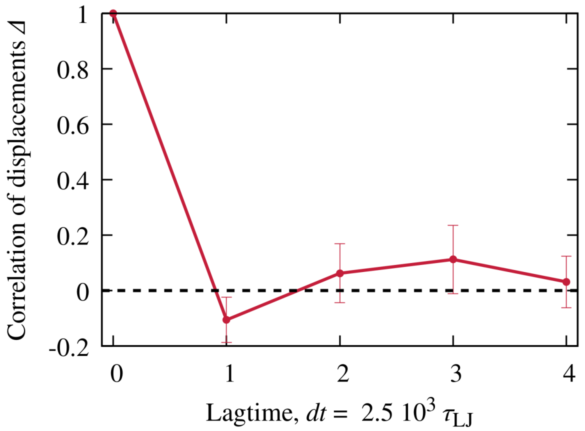

FIG. S12. **Time correlation function of guest-ring displacements.** The autocorrelation function of guest ring displacements, x,computed from trajectory excerpts with x in $[6;16]$ (flat free-energy region), become practically negligible in a single sampling step, consistent with the overdamped nature of the Wiener process.

RING COMPENETRATION IN COLLAPSED CATENANES

Threadings are, at least in part, facilitated by the substantial compenetration of concatenated rings. To quantify the effect we computed monomer (bead) density in spherical shells of thickness $\delta r = 0.25\sigma$ at increasing radial distance, r, from the rings' centers of mass of catenanes $n = 50$ rings of $m = 40$ beads, and averaging it over the rings. The

density is shown in panel (a) with a pile-up graph, highlighting the contribution of monomers from the same ring, from neighboring (concatenated) rings and from other rings. The graph in panel (b) shows the number of beads in the shells, i.e. corresponding to the data of panel (a) multiplied by the shell volume.

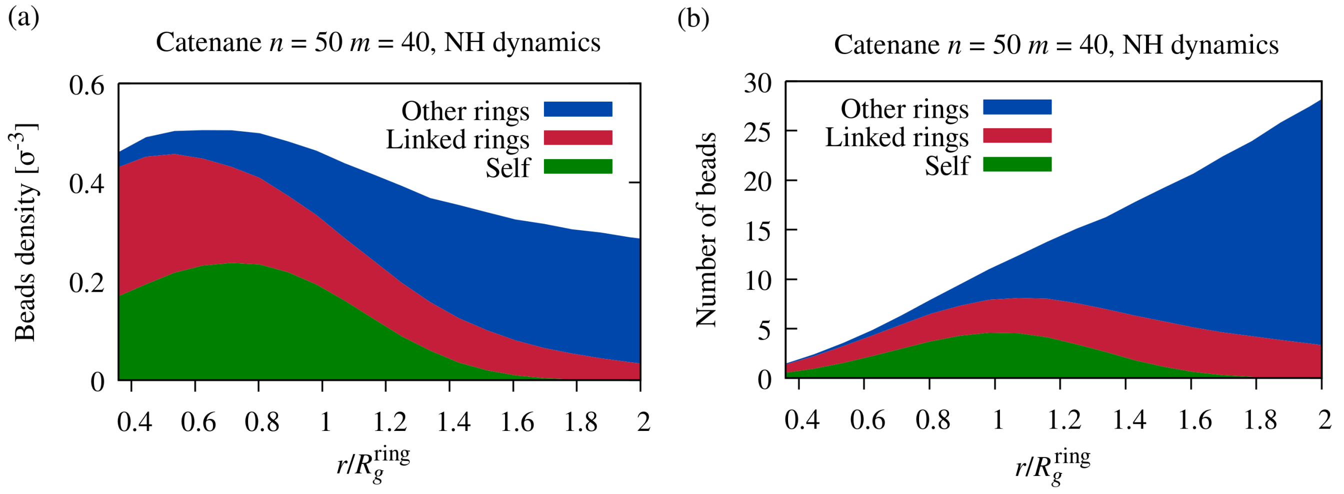

FIG. S13. **Monomer density in collapsed catenanes**. a) Pile-up graph of the monomer (bead) density in spherical shells of thickness $\delta r = 0.25\sigma$ at increasing radial distance, r, from the rings' centers of mass of catenanes $n = 50$ rings of $m = 40$ beads, averaged it over the rings. The pile-up graph, highlights the contribution of monomers from the same ring, from neighboring (concatenated) rings and from other rings. The graph in panel (b) shows the number of beads in the shells.

BRANCHED POLYMER CHAIN AND POSITIONAL BIASES OF TERMINI AND BRANCHING POINTS

We considered collapsed branched chains-of-beads to illuminate the observed bias of terminal and threaded rings to be, respectively, at the surface and core of the collapsed catenanes. The correspondence is motivated by the observation that threadings can be viewed as branching points for the catenanes' backbone.

We thus considered a branched linear polymer, with a main chain of 100 beads and two side chains of 5 beads each. The branching points are at one quarter of the backbone contour (bead 25). We measured the burial degree of the 4 terminal points (main chain and side chains) and of the branching point in the collapsed state, obtaining the distributions shown in Fig. S14.

The results show that the termini, the monomers with lowest connectivity degree, are exposed, while the branching point, which has the highest connectivity degree is buried. These different burial propensities match those observed in catenanes, thus clarifying that they mostly originate from the different effective connectivity of terminal and threaded rings, and not due to other features specific to catenanes.

REFERENCES

[1] S. Plimpton, Journal of computational physics **117**, 1 (1995).
[2] K. Kremer and G. S. Grest, The Journal of Chemical Physics **92**, 5057 (1990).
[3] P. Chiarantoni and C. Micheletti, Macromolecules **55**, 4523 (2022).
[4] L. Tubiana, G. Polles, E. Orlandini, and C. Micheletti, The European Physical Journal E **41**, 1 (2018).
[5] K. Koniaris and M. Muthukumar, The Journal of chemical physics **95**, 2873 (1991).
[6] C. Micheletti, G. Bussi, and A. Laio, The Journal of Chemical Physics **129** (2008), 10.1063/1.2969761.

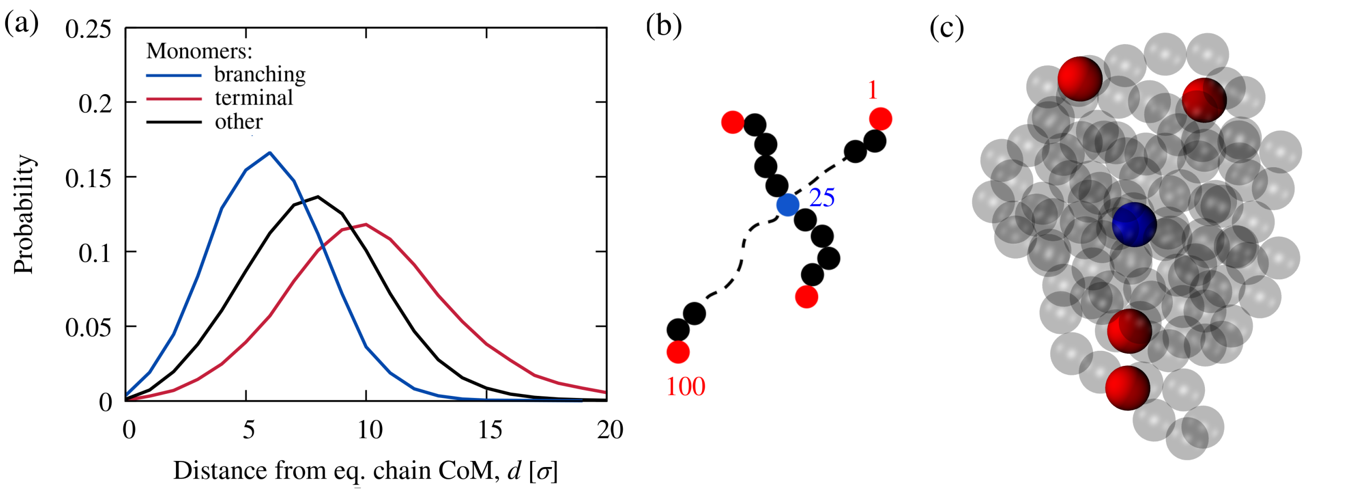

FIG. S14. **Probability distributions of distances from the globule CoM, sketch and snapshot for a branched linear chain.** (a) As the connectivity of the bead increases, the distributions are shifted towards smaller values of d, indicating that while the ends tends to be on the surface, a branching point tends to remain more inside the globule, thus explaining the entropic barrier that hinders unthreading via the end mode. (b) Sketch of the branched linear chain, with four terminal monomers (red, connectivity 1), one branching monomer (blue, connectivity 4) and 105 monomers in the chain (black, connectivity 2). (c) Snapshot of the branched polymer in the collapsed state where the positional biases of the termini and the branching point are visible.
